# The Aryl Hydrocarbon Receptor and the Maintenance of Lung Health

**DOI:** 10.3390/ijms19123882

**Published:** 2018-12-05

**Authors:** Necola Guerrina, Hussein Traboulsi, David H. Eidelman, Carolyn J. Baglole

**Affiliations:** 1 Research Institute of the McGill University Health Centre, Montreal, QC H4A 3J1, Canada; necola.guerrina@mail.mcgill.ca (N.G.); hussein.traboulsi@mail.mcgill.ca (H.T.); david.h.eidelman@mcgill.ca (D.H.E.); 2 Department of Pathology, McGill University, Montreal, QC H3A 2B4, Canada; 3 Department of Medicine, McGill University, Montreal, QC H4A 3J1, Canada; 4 Department of Pharmacology and Therapeutics, McGill University, Montreal, QC H3G 1Y6, Canada

**Keywords:** aryl hydrocarbon receptor, lung, cigarette smoke, chronic obstructive pulmonary disease

## Abstract

Much of what is known about the Aryl Hydrocarbon Receptor (AhR) centers on its ability to mediate the deleterious effects of the environmental toxicant 2,3,7,8-tetrachlorodibenzo-p-dioxin (TCDD; dioxin). However, the AhR is both ubiquitously-expressed and evolutionarily-conserved, suggesting that it evolved for purposes beyond strictly mediating responses to man-made environmental toxicants. There is growing evidence that the AhR is required for the maintenance of health, as it is implicated in physiological processes such as xenobiotic metabolism, organ development and immunity. Dysregulation of AhR expression and activity is also associated with a variety of disease states, particularly those at barrier organs such as the skin, gut and lungs. The lungs are particularly vulnerable to inhaled toxicants such as cigarette smoke. However, the role of the AhR in diseases such as chronic obstructive pulmonary disease (COPD)—a respiratory illness caused predominately by cigarette smoking—and lung cancer remains largely unexplored. This review will discuss the growing body of literature that provides evidence that the AhR protects the lungs against the damaging effects of cigarette smoke.

## 1. Introduction

The Aryl Hydrocarbon Receptor (AhR) is a ligand-activated transcription factor that is evolutionarily-conserved among multicellular organisms [1]. The expression and distribution of AhR was recently reviewed [2]. Although ubiquitously-expressed, the level of AhR expression is variable. For example, the highest expression of the AhR occurs in first-line defense organs such as the lungs, skin, liver and gut [2]. The AhR belongs to the basic helix loop helix (bHLH)/PER-ARNT-SIM (PAS) family whose members are involved in the sensing of exogenous and endogenous stimuli [3]. The AhR belongs to the bHLH superfamily marked by the presence of a basic domain and a HLH domain. The basic domain enables binding of the AhR to DNA, whereas the HLH domain enables protein dimerization [4]. This superfamily has been further subdivided phylogenetically into six classes (A-F). The AhR belongs to bHLH class C, as it both recognizes and binds ACGTG or GCGTG DNA sequences and contains a PAS domain [4]. The bHLH and PAS domains are located on the amino-terminus of the AhR protein, whereas the transactivation domain is found on the carboxy-terminus and influences the nucleo-cytoplasmic shuttling functions of the amino-terminus [5].

Conventionally, the AhR is activated by a wide variety of agonists of either exogenous or endogenous origin. These ligands are typically hydrophobic in nature and thus enter the cell via simple diffusion through the plasma membrane [6]. AhR ligands are also classically small (i.e., estimated to be between 1.2 and 1.4 nm in length) and planar substances [7], although structurally-diverse ligands that deviate from these properties have been recently identified [8]. Even a complex mixture such as cigarette smoke (CS) is an AhR agonist, inducing significant AhR activation in vitro [9,10] and in vivo [11,12]. CS contains more than 5000 chemicals, including metals (iron, nickel), gases (ozone), biological agents (plant pollen, endotoxins, bacteria), minerals (quartz, asbestos) and organic chemicals such as nicotine, polychlorinated dibenzodioxins [PCDDs] and polycyclic aromatic hydrocarbons [PAH] [13,14,15,16]. Many of the individual organic chemicals found in CS are also ligands of the AhR [11,17]. Although the AhR may mediate carcinogenicity induced by individual components of CS, such as 2,3,7,8-tetrachlorodibenzo-p-dioxin (TCDD) [18] and benzo[a]pyrene [19], it simultaneously protects against many of the pathogenic effects of CS in the lung [9,10,11,12,20]. This demonstrates that CS is more than simply the sum of its parts and that CS represents an AhR agonist that is structurally and functionally distinct from the individual constituents of CS.

Cigarette smoking is the primary cause of prevalent respiratory pathologies such as chronic obstructive pulmonary disease (COPD) and lung cancer, contributing to approximately 75% of both COPD [21] and lung cancer [22] cases. The pathogenesis of these diseases involves the deregulation of cellular processes including cell survival, migration and proliferation. These cellular processes are also controlled by the AhR [23,24,25,26]. It remains controversial however, as to the extent to which aberrant AhR expression and/or activation contributes to disease. For example, over-expression of the AhR occurs in many types of human cancers, including lung, breast and gastric cancers [25,27,28,29,30] and there is recent evidence supporting a pro-oncogenic role for AhR over-expression in lung cancer [31]. Whether the AhR contributes to the pathogenesis of COPD remains unexplored. The focus of this review is to highlight the growing body of literature that demonstrates a role for the AhR in attenuating CS-induced lung injury, with particular emphasis on COPD.

## 2. The Aryl Hydrocarbon Receptor (AhR)

### 2.1. AhR Ligands

#### 2.1.1. Exogenous AhR Agonists

The best characterized class of AhR ligands are exogenous environmental toxicants, including PAHs and halogenated aromatic hydrocarbons (HAHs) [7]. Both PAHs and HAHs are produced from the incomplete combustion of organic compounds, which can occur naturally (e.g., forest fires and volcanic eruptions) or from anthropogenic sources (e.g., burning of fossil fuels, pulp and paper manufacturing, air pollution, CS) [8]. Exposure to PAHs and HAHs typically induces the expression of xenobiotic metabolizing enzymes (XMEs), which are target genes of the AhR. Although these XMEs are important in the metabolism and clearance of most PAHs [8], XMEs are often ineffective in the metabolism of many HAHs-particularly chlorinated HAHs. Chlorinated HAHs, also known as dioxin-like compounds, can be sub-divided into three classes: polychlorinated biphenyls (PCBs), polychlorinated dibenzofurans (PCDFs) and polychlorinated dibenzodioxins (PCDDs). 

The best known AhR ligand is TCDD, which belongs to the PCDD class. TCDD is a colorless and odorless solid whose chemical structure is characterized by the presence of a central oxygenated ring surrounded by two benzene rings. TCDD has one of the highest binding affinities of known AhR ligands- in the pM to nM range [2]. TCDD toxicity is a consequence of its chlorine substituents being located in lateral positions (on carbons 2, 3, 7 and 8), resulting in impaired metabolism and clearance. Although the exact duration of TCDD persistence will vary as a consequence of dose, exposure duration and body composition [32], the half-life of TCDD in humans ranges from approximately 7–12 years [33,34]. There have been a number of incidences whereby humans have been exposed to TCDD, including those related to occupations involving chemical and pesticide production. Accidental environmental contaminations to petrochemicals and industrial waste have also been reported [35]. Two well-characterized examples of human exposure to TCDD come from individuals subjected to the herbicide Agent Orange during the US-Vietnam war [36] and from the poisoning of the former Ukrainian President Viktor Yushenko [37]. Hallmark clinical manifestations of TCDD-induced toxicity in humans are hepatotoxicity; epidermal symptoms including chloracne and hyperpigmentation; cancers of the breast, lung and liver as well as immunosuppression [38]. Findings in animal models indicate that these deleterious health effects are mediated by the AhR [18,39,40].

In addition to PAHs and HAHs, several classes of naturally-occurring exogenous AhR ligands have been recently described, including bacterial products and compounds derived from a healthy diet. Food-derived AhR ligands typically have a lower affinity for the AhR compared to TCDD, within the µM to mM range [2]. Several classes of dietary AhR agonists include indole-3-carbinol derivatives found in leafy greens such as broccoli and Brussels sprouts, as well as flavonoids found in fruits and vegetables [7]. Finally, one of ways through which the AhR contributes to the defense against bacterial infection is by sensing bacterial products (i.e., virulence factors) from *Pseudomonas aeruginosa* [*P. aeruginosa*] and *Mycobacterium tuberculosis* [*M. tuberculosis*], which function as AhR agonists [41].

#### 2.1.2. Endogenous AhR Agonists

In addition to exogenous AhR agonists, there are several classes of endogenous AhR agonists. These include heme derivatives such as bilirubin and biliverdin and arachidonic acid metabolites such as prostaglandins and leukotrienes [2,7]. Although several prostaglandins have been reported to activate the AhR (PGB_2_, PGD_2_, PGF_3α_, PGG_2_, PGH_1_ and PGH_2_), prostaglandins and heme-derivatives are considered low-affinity ligands because they require µM concentrations to induce AhR activation [2,7]. In contrast, the arachidonic acid metabolite lipoxin A_4_ binds to the AhR and induces significant AhR activation in nM concentrations [42]. 

Tryptophan metabolites are another class of endogenous AhR agonists. Two well-characterized members of this class are kynurenine and 6-formylindolo (3,2-b) carbazole (FICZ). Kynurenine is produced during the first step of tryptophan metabolism, mediated by indoleamine 2,3-dioxygenase (IDO) [43], whereas FICZ is a tryptophan-derived ultraviolet (UV)-photoproduct [7]. Another endogenous AhR ligand that was first described in the pig lung is 2-(1′H-indole-3′-carbonyl)-thiazole-4-carboxylic acid methyl ester (ITE) [7]. Kynurenine (and its metabolites), FICZ and ITE are high affinity endogenous agonists that cause AhR activation in pM to nM concentrations [2,7,44]. 

### 2.2. AhR Signaling Pathways

#### 2.2.1. Canonical AhR Signaling 

In the absence of ligands, the AhR is located in the cytoplasm and stably complexed with a heat shock protein 90 (HSP90) homodimer, immunophilin-like X-associated protein 2 (XAP2) and the co-chaperone p23 [7,45]. In this core complex, HSP90 ensures proper folding of newly-synthesized AhR protein [46]. XAP2 (also known as ARA9 or AhR-interacting protein [AIP]) stabilizes the unligated AhR, resulting in heightened AhR expression and transcriptional activity [46]. The role of p23 is to prevent degradation of the unligated AhR, as supported by findings that a down-regulation of p23 results in increased ubiquitination and proteasomal degradation of the AhR protein [47]. Thus, the cumulative effect of this AhR complex is to stabilize the cytoplasmic AhR protein.

**Figure 1:**
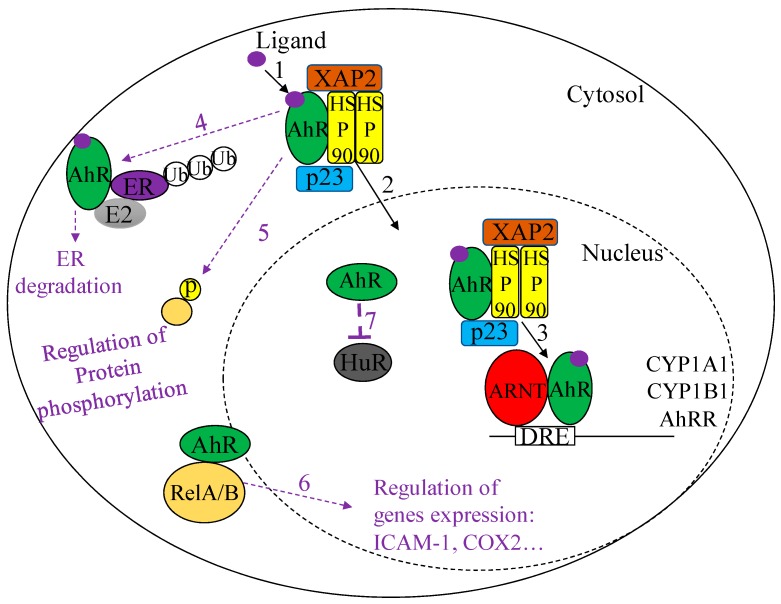
Diagrammatic representation of Aryl hydrocarbon receptor (AhR) signaling pathways. Canonical AhR Signaling (solid black arrows)-Inactivated AhR is found in the cytosol (solid circle) complexed to a HSP90 homodimer, XAP2 and p23. (1) Ligand binding to the AhR induces the (2) translocation of the AhR complex from the cytoplasm to the nucleus (dashed circle). In the nucleus the AhR dissociates from its chaperone proteins, (3) heterodimerizes with ARNT and binds to DRE sequences to induce transcription of target genes. Non-canonical AhR Signaling (dashed purple arrows)-The AhR can heterodimerize with a variety of proteins to mediate diverse downstream effects. (4) When complexed with the estrogen receptor (ER), the AhR functions as an E3-ligase to mediate ER ubiquitination and degradation. (5) The AhR also mediates phosphorylation events, such as the phosphorylation of Akt and ERK proteins in the cytoplasm. (6) The AhR can also bind to the NF*κB* family members RelA and RelB, where it can regulate the expression of pro-inflammatory mediators such as IL6, COX-2 and ICAM1. (7) The AhR can also regulate the subcellular localization of RNA-binding proteins such as HuR, thereby indirectly influencing mRNA stability and thus expression of pro-inflammatory mediators such as COX-2.

When in the cytoplasm, AhR ligands bind to the ligand-binding domain located on the amino terminus of the AhR protein. After ligand binding, the AhR core complex translocates from the cytoplasm to the nucleus. This ligand-induced AhR nuclear translocation is mediated by the dephosphorylation of two protein kinase C sites found on serine 12 and 36, which are located upstream of the AhR nuclear localization signal (NLS) [48,49]. In the nucleus, the AhR dissociates from its chaperone proteins [50,51] prior to heterodimerization with the aryl hydrocarbon receptor nuclear translocator (ARNT). This AhR-ARNT complex binds to the dioxin response element (DRE), also known as the xenobiotic response element (XRE) or the aromatic hydrocarbon response element (AhRE) (Figure 1). The DRE has a core sequence of 5′-TNGCGTG-3′ and is located in the promoter region of AhR target genes, the prototype of which is cytochrome P450 (CYP) CYP1A1 [52]. There are eight DREs located within the murine CYP1A1 promoter [52]. Once the AhR-ARNT complex is bound to the DRE, transcriptional co-activators facilitate the recruitment of RNA polymerase II to this complex, resulting in the initiation of transcription of AhR target genes [53]. Following the transcriptional induction of AhR target genes, the AhR dissociates from ARNT and is shuttled back to the cytoplasm via unmasking of its nuclear export signal [48]. The AhR protein is then degraded by the 26S ubiquitin-proteasome system [54]. Activation of the AhR also initiates the transcription of the AhR repressor (AhRR), which functions as a negative regulator of AhR activity. The AhRR can negatively regulate AhR activity by directly interfering with AhR-ARNT interactions, which reduces AhR activity and facilitates AhR protein degradation [55]. In addition to the AhRR, TCDD-inducible Poly ADP-Ribose Polymerase (TiPARP) is another AhR target gene that functions to negatively regulate AhR activity by modulating AhR expression. This is supported by experimental evidence that TiPARP over-expression results in heighted proteolytic degradation of the AhR [56]. 

#### 2.2.2. Non-Canonical AhR Signaling

The toxic effects of the high-affinity AhR ligand TCDD are widely understood to be mediated through the canonical AhR pathway [39,40]. This pathway is characterized by (1) ligand binding to the AhR, (2) AhR nuclear translocation, (3) AhR-ARNT heterodimerization and (4) AhR binding to the DRE to induce transcription of target genes. Paradoxically, activation of the AhR by the high-affinity AhR ligands FICZ and ITE yield non-toxic effects [57,58]. One proposed explanation for differential outcomes by AhR ligands is the activation of alternative AhR signaling mechanisms, which are often collectively referred to as non-canonical AhR signaling pathways. One example of non-canonical AhR signaling is regulation of fibroblast proliferation, which may occur through a ligand-independent mechanism. Here, AhR-expressing cells-that have higher proliferation rates than AhR-deficient cells [59]-is thought to be ligand-independent because proliferation rates in AhR-expressing cells are unaffected by deletion of the AhR ligand-binding domain [59]. Furthermore, even when the ligand binding domain is absent and ligand binding to the AhR cannot occur, the AhR can still both dimerize with ARNT and induce transcription of AhR target genes [60]. 

Non-canonical AhR signaling can also occur through direct physical interactions with proteins not typically required for the canonical AhR pathway. The AhR can form dimers with a variety of proteins that are involved in the cell cycle, apoptosis and the immune response [61]. For example, AhR activation by TCDD initiates a physical interaction between AhR and the estrogen receptor (ER) [62,63], whereby the AhR functions as an E3-ligase and mediates ER degradation [64,65]. Such AhR-dependent ER degradation demonstrates how exposure to TCDD can modulate sex hormone expression. Additionally, by forming a protein complex with the signal transducer and activator of transcription 1 (STAT1), the AhR attenuates lipopolysaccharide (LPS)-induced expression of the pro-inflammatory cytokine interleukin-6 (IL-6) in macrophages [66]. This AhR-STAT1 complex suppresses IL-6 promoter activity and thus reduces IL-6 production [66]. Finally, the AhR physically interacts with the nuclear factor kappa beta (NF-κB) protein RelA, resulting in increased cellular proliferation and tumorigenesis [27]. In lung cancer cells, AhR-RelA dimers bind to a DRE-independent NF-κB response element in the IL-6 promoter, resulting in increased IL-6 expression [28]. 

Lastly, the AhR also signals non-canonically by regulating phosphorylation events in either the cytoplasm or the nucleus. For example, the AhR mediates TCDD-induced phosphorylation of Akt and ERK [67]. This phosphorylation is unaffected by ablation of the AhR NLS, suggesting that this occurs in the cytoplasm [67]. However, the AhR also promotes phosphorylation events in the nucleus. For example, the AhR-mediates the recruitment of the inhibitor of nuclear factor kappa beta kinase subunit alpha (IKKα) to the promoter of AhR target genes, resulting in the phosphorylation of histone 3 at serine 10 (H3S10) within the DNA [68]. This phosphorylation event is required for TCDD-induced upregulation of AhR target genes such as *Cyp1a1* [68]. Both the AhR-mediated recruitment of IKKα, and the subsequent phosphorylation event, represent non-genomic roles for the AhR that ultimately contribute to enhanced transcriptional (i.e., canonical) AhR signaling. This highlights the inter-related nature of the various modes of AhR signaling. 

### 2.3. Physiological Functions of the AhR

Initiation of an AhR signaling pathway(s) is important for the regulation of diverse downstream physiological processes. Several of these processes include xenobiotic metabolism, organ development and immunity.

#### 2.3.1. Xenobiotic Metabolism

The AhR promotes the upregulation of many XMEs. The first-identified members of the AhR gene battery are XMEs including CYP1A1, CYP1A2, NAD(P)H quinone oxidoreductase 1 (NQO1), aldehyde dehydrogenase (ALDH) ALDH3a1, UDP-glucuronosyltransferase (UGT) UGT1a6 and glutathione s-transferase (GST) GSTa1 [69]. XMEs render hydrophobic substances water soluble to facilitate their elimination. In addition to xenobiotics, XMEs also metabolize endogenous substances such as eicosanoids, including prostaglandins and leukotrienes [70]. 

The CYP enzymes are Phase I XMEs that catalyze the addition, or the unmasking, of a polar functional group (e.g., -OH, -SH, -NH_2_), which can result in the formation of an active metabolite. Induction of CYP enzymes, especially CYP1A1, is typically used as evidence of AhR activation [71,72]. NQO1, ALDH3a1, UGT1a6 and GSTa1 are Phase II XMEs [73]. Phase II reactions increase water solubility of the xenobiotic via the conjugation of polar subgroups, leading to the formation of an inactive metabolite that is subsequently excreted [74]. Small lipophobic metabolites are typically excreted via the kidney into the urine, whereas larger and more lipophilic metabolites are preferentially excreted by the liver into the bile and subsequently into fecal matter [75]. Although not the predominant route of excretion, many xenobiotics (particularly gases, vapors and highly lipophilic xenobiotics) are eliminated via the lungs [76]. 

#### 2.3.2. Organ Development 

The AhR is necessary for proper organ development. AhR-deficient mice have reduced liver weight and hepatic ductal fibrosis [77]. Similarly, AhR activity in both endothelial and hematopoietic cells is necessary for vascular development and the closure of the ductus venosus [78]. The ductus venosus is a fetal structure that typically closes after birth and serves to redirect oxygenated blood coming from the placenta away from the fetal liver and towards the inferior vena cava of the fetal heart. Persistence of a patent ductus venosus after birth is associated with liver disease in adults [79]. Both AhR-deficient and TCDD-exposed AhR-expressing mice exhibit defects in cardiac development and function [80], suggesting that homeostatic regulation of AhR expression and/or activity is necessary for proper cardiac development. 

The AhR may also contribute to the development of the central nervous system (CNS), as recently reviewed in this Journal [81]. AhR-deficient *Caenorhabditis elegans* (*C. elegans*) exhibit impairments in neuronal migration, differentiation and axonal branching [82]. Additionally, AhR ablation also results in cognitive defects in the murine hippocampus leading to reduced fear memory [83] and abnormal ocular movements [84]. Visual impairments observed in AhR-deficient mice may be a consequence of optic nerve demyelination and thus defective signal transduction. AhR ablation causes altered myelin composition and an increased proportion of demyelinated axons in the optic nerve [85]. Interestingly, models utilizing constitutively active AhR demonstrate that over-activation of the AhR elicits outcomes similar to AhR deficiency, as constitutive AhR activation also impairs neuronal migration in both the hippocampus and olfactory bulb [86,87]. It appears that homeostatic levels of AhR expression/activity are also critical for CNS development. 

#### 2.3.3. Immunity

The AhR protects organs from foreign invaders via its influence in both the innate and adaptive branches of the immune system. In the context of the innate immune system, the AhR promotes endotoxin tolerance, thereby dampening the pathologic effects of infection on the host [88]. The AhR also regulates different branches of adaptive immunity. In the humoral arm of the adaptive immune system, the AhR is critical for optimal levels of both B-cell proliferation [23] and differentiation (i.e., differentiation into antibody-secreting plasma B-cells versus memory B-cells) [89]. In respect to T-cell mediated immunity, the AhR controls the differentiation of both helper T lymphocytes (Th17) and regulatory T lymphocytes (Tregs) through both direct and indirect mechanisms in a context specific manner [90]. One direct mechanism may be via methylation changes. This is supported by a report demonstrating that an AhR-mediated promotion of Treg differentiation and prevention of Th17 differentiation is mechanistically mediated by *Foxp3* promoter demethylation and IL-17 promoter hypermethylation, respectively [91]. An indirect mechanism through which the AhR may influence Th17 levels is via the regulation of the gut microbiome. For example, AhR-deficient mice have an increased abundance of *Verrucomicrobiota* and segmented filamentous bacteria in the gut, which is directly linked to elevated intestinal inflammation and heightened IL17a expression, suggesting a promotion of Th17 differentiation [92]. The differential outcome of Treg versus Th17 cell differentiation is also controlled by different AhR ligands. For example, kynurenine causes AhR-dependent production of Tregs whereas AhR activation by FICZ leads to the production of Th17 cells [43].

## 3. The AhR in CS-Induced Lung Disease

### 3.1. Lung Cancer

Lung cancer is the leading cause of cancer-related deaths worldwide (WHO). The role of the AhR in the promotion of lung cancer pathogenesis was recently reviewed [31], therefore it will only be briefly discussed here. Lung cancer is associated with AhR over-expression [29]. Over-expression or constitutive activation of AhR in cancer cell lines promotes tumor growth, whereas AhR inhibition in cancer cells leads to reduced cell proliferation and migration [30]. A pro-oncogenic role for AhR over-expression in lung cancer may be a consequence of an AhR-dependent promotion of cell proliferation [23,24], cell-cycle progression, cell migration [25] and cell survival [26]. Mechanistically, the AhR-dependent promotion of several pro-oncogenic signaling pathways (e.g., JAK2-Src interaction) is independent of the transcriptional activities of the AhR, suggesting the involvement of non-canonical AhR signaling in the promotion of lung cancer progression [67].

### 3.2. Chronic Obstructive Pulmonary Disease (COPD)

COPD is a respiratory illness characterized by progressive and irreversible air flow obstruction. Worldwide, COPD affects an estimated 380 million people [93] and is the third leading cause of death. Although often referred to as a “disease”, COPD encompasses a spectrum of disorders with two predominant phenotypes: chronic bronchitis and emphysema. Chronic bronchitis predominantly affects the airways and is characterized by mucus hypersecretion, which functionally leads to airway obstruction and a productive cough [94]. In contrast, emphysema is an anatomical condition characterized by the permanent destruction of the alveolar walls, resulting in parenchymal destruction [95,96]. Despite the fact that chronic bronchitis and emphysema can present independently of one another, it is now widely accepted that most cases of COPD typically fall somewhere in the middle of a “COPD-spectrum” and individuals with COPD often exhibit characteristics of both chronic bronchitis and emphysema to varying extents [94].

Although COPD is predominantly caused by CS, other environmental risk factors include inhalational exposure to ambient (e.g., air pollution) and occupational (e.g., coal mines and pulp and paper manufacturing) toxicants [97,98,99,100,101]. Moreover, only 15–20% of smokers go on to develop COPD, indicating that factors beyond exposure to inhalational toxicants are important. This includes genetic factors [102,103]. The only established genetic risk factor for COPD is alpha-1 antitrypsin (AAT) deficiency [104], which occurs in 3–10% of individuals with COPD [105,106]. However, COPD is a heterogenous disease with many interrelated pathogenic mechanisms including inflammation, oxidative stress and cell death; there is also a growing body of experimental evidence demonstrating that the AhR attenuates several of these mechanisms that ultimately contribute to the development of this disease.

#### 3.2.1. Inflammation

Cigarette smoking promotes pulmonary inflammation in that the number and proportion of immune cells in the lung shifts in response to CS exposure. Human cigarette smokers have heightened levels of pulmonary neutrophils, macrophages and CD8^+^ T-lymphocytes [107]. These cell types are also increased in mice exposed to CS [108]. In COPD subjects, the quantity of these cell types are further increased compared to smokers without COPD [109]. Although macrophages and CD8^+^ T-lymphocytes are the predominant inflammatory cell types in the lungs of humans with COPD, neutrophilia is also common [110]. Elevated neutrophil numbers are also seen in the bronchoalveolar lavage (BAL) of mice exhibiting a COPD-like phenotype [111]. 

Neutrophils are the first immune cell type recruited to the lung in response to CS, exhibiting a significant increase in the BAL after only 3-days of CS exposure [108]. AhR-deficient mice exhibit significantly greater neutrophilia than AhR-expressing mice following both an acute (3 day) [112] and extended (2–4 week) CS exposure [11,12], indicating a role for the AhR in attenuating this early CS-induced neutrophilic response. The mechanism by which this occurs is incompletely understood. One possibility involves the AhR-dependent regulation of the NF-κB protein RelB. The AhR physically interacts with RelB [113,114]. Moreover, AhR-deficient mice exhibit a more rapid degradation of pulmonary RelB protein following CS exposure than that observed in CS-exposed AhR-expressing mice [112]. Increased RelB degradation in AhR-deficient mice is associated with increased expression of the neutrophil adhesion protein ICAM1 and heightened neutrophil infiltration [112]. These findings suggest the possibility that the AhR may attenuate CS-induced neutrophilia via a non-canonical protein interaction with RelB. 

CS-induced macrophage and lymphocyte pulmonary infiltration is delayed relative to neutrophils. Significant increases of macrophage and lymphocyte numbers in the murine BAL occurs after approximately 10-days of CS exposure, whereas significant increases in the lung parenchyma is only observed after several months of CS exposure [108]. The longest in vivo CS exposure that has been reported using AhR-deficient and AhR-expressing mice is 4-weeks [11]. Based on this study, it appears that CS-induced macrophage and lymphocyte infiltration in the murine lung is independent of the AhR because although this CS duration induces a significant increase in the number of BAL macrophages and lymphocytes, this increase is independent of AhR expression [11]. 

The pro-inflammatory enzyme cyclooxygenase-2 (COX-2) is robustly induced in response to CS exposure [115] and is elevated in COPD subjects [10]. COX-2 may contribute to COPD pathogenesis because it initiates the downstream production of several classes of inflammatory mediators such as prostacyclins, prostaglandins (PG) and thromboxanes [116]. Experimentally, inhibition of COX-2 expression (and the downstream COX-2 product PGE_2_) using celecoxib attenuates the development of CS-induced airspace enlargement in the rat lung [117]. The AhR-mediated regulation of RelB may attenuate other aspects of CS-induced inflammation, such as the production of COX-2. In support of this, RelB reduces the expression of COX-2 [115]. Another non-canonical AhR-protein interaction that may contribute to the AhR attenuation of CS-induced inflammation is its regulation of Human Antigen R (HuR). HuR is an RNA-binding protein that functions to stabilize target mRNA when localized to the cytoplasm, thereby promoting mRNA translation into protein. *Cox2* is a target mRNA of the HuR [10]. The AhR attenuates CS-induced COX-2 expression via the nuclear sequestration of HuR, resulting in the destabilization and degradation of *Cox2* mRNA in vitro [10]. This resulted in reduced *Cox2* mRNA and protein expression in AhR-expressing cells [10]. The AhR may therefore represent a physiological regulatory mechanism to limit COX-2 overexpression. This is supported by evidence that COX-2 products, such as prostaglandins, act as AhR agonists [118] and the activated AhR then functions to attenuate CS-induced COX-2 production [10].

#### 3.2.2. Oxidative Stress 

Oxidative stress is another mechanism linked to COPD pathogenesis [119]. In the healthy lung, reactive oxygen species (ROS)- such as superoxide anions, hydroxyl radicals and hydrogen peroxide- are counterbalanced by the production of endogenous antioxidants, including superoxide dismutase (SOD), catalase (CAT) and the glutathione (GSH)/glutathione peroxidase system [120]. When ROS production exceeds the capabilities of these antioxidant defenses, oxidative stress ensues. Inhalational exposure to CS results in heightened ROS production in the lungs, as approximately 10^17^ oxidant molecules are produced with each puff of a cigarette [121]. Additionally, ROS production by recruited immune cells (e.g., neutrophils and macrophages) represent another major oxidant source [120]. Cigarette smokers exhibit oxidative stress-induced lipid peroxidation [122] and reduced antioxidant capacities (e.g., reduced SOD and CAT activity [123]). There is evidence to support that AhR attenuates CS-induced oxidative stress. CS-exposed AhR-deficient lung structural cells exhibit significantly higher ROS production compared to CS-exposed AhR-expressing cells [9]. Additionally, AhR-deficient mouse lung fibroblasts (MLFs) also exhibit an impaired induction of the antioxidants sulfiredoxin 1 (*Srxn1*) and NADPH: quinone acceptor oxidoreductase 1 (*Nqo1*) following in vitro exposure to CS relative to AhR-expressing MLFs [9]. 

However, factors other than strictly CS likely contribute to the oxidative stress observed in COPD. In support of this, greater lipid peroxidation is observed in individuals with COPD that have never smoked relative to subjects without COPD [124]. Moreover, COPD subjects also have significantly reduced antioxidant expression (e.g., SOD and GSH levels) relative to smokers without COPD [125]. Reduced AhR expression in COPD-derived lung structural cells [9] has the potential to increase oxidative stress in the COPD lung. Consistent with this notion, AhR-deficient MLFs exhibit reduced expression of the antioxidants SOD1 and SOD2 relative to AhR-expressing MLFs at baseline [20]. This illustrates that AhR ablation is associated with a diminished expression of antioxidants that are necessary to combat oxidative stress. 

#### 3.2.3. Loss of Lung Structural Cells

A hallmark of the emphysema component of COPD is the loss of lung structural cells [126,127,128]. This includes loss of alveolar epithelial cells responsible for gas exchange and fibroblasts that synthesize the extracellular matrix necessary for lung structure and elasticity. CS induces apoptotic cell death in all major lung structural cell types, including bronchial and alveolar epithelial cells, fibroblasts, endothelial cells and airway smooth muscle cells [129,130,131,132,133]. Furthermore, humans with emphysema exhibit heightened pulmonary apoptosis [134]. Experimentally, intra-tracheal injection of the apoptotic protein cleaved caspase-3 induces epithelial cell apoptosis and airspace enlargement in the murine lung [126], consistent with the notion that lung parenchymal destruction is linked to cell death. 

The AhR has the potential to contribute to loss of structural cells in several ways. CS-exposed AhR-deficient lung structural cells exhibit significantly more apoptosis [20]. Mechanistically, attenuation of CS-induced apoptosis in vitro is mediated by the AhR-dependent regulation of miR-196a [135]. Another mechanism may be that loss of lung structural cells in the COPD lung is due to decreased replacement of lost cells. AhR-deficient cells exhibit reduced cellular proliferation relative to AhR-expressing cells [136]. This is similar to the reduced proliferative capacity of lung fibroblasts from subjects with emphysema [137,138]. Cellular senescence may also contribute to loss of lung structural cells in COPD, as it is increased in alveolar epithelial cells from subjects with emphysema and is also positively correlated with more severe airflow obstruction [139]. Interestingly, cultured AhR-deficient mouse embryonic fibroblasts (MEFs) reach a state of senescence more rapidly than AhR-expressing MEFs [140]. Collectively, these findings suggest that the AhR may attenuate lung parenchymal destruction by promoting conditions that reduce the loss of lung structural, including altering cell death, proliferative capacities and/or cellular senescence. 

#### 3.2.4. Exacerbations

Another important clinical feature of COPD is the occurrence of exacerbations, which are bouts of worsened symptoms that negatively impact quality of life [141]. COPD exacerbations are largely believed to be from infectious agents [142], particularly bacterial in origin [143]. The AhR is implicated in attenuating several bacterial lung infections of relevance to COPD exacerbations. Two of the most commonly-isolated bacteria associated with COPD exacerbations are *Streptococcus pneumoniae* (reported in 10–15% of cases) and *P. aeruginosa* (reported in 5–10% of cases) [144]. Experimental evidence has demonstrated that following challenge with *S. pneumoniae*, AhR activation by the exogenous ligand TCDD reduces lung bacterial burden and increases survival [145]. The AhR also helps maintain host defense against *P. aeruginosa*. AhR-deficiency results in increased lung bacterial burden and reduced mortality following *P. aeruginosa* infection relative to AhR-expressing mice [41]. Mechanistically, the AhR may contribute to a coordinated immune response against *P. aeruginosa* by sensing bacterially produced virulence factors (e.g., pyocyanin), which act as AhR agonists to induce an AhR-mediated production of the neutrophil chemoattractant IL-8 [146] and the subsequent recruitment of neutrophils [41]. 

## 4. Conclusions

**Figure 2:**
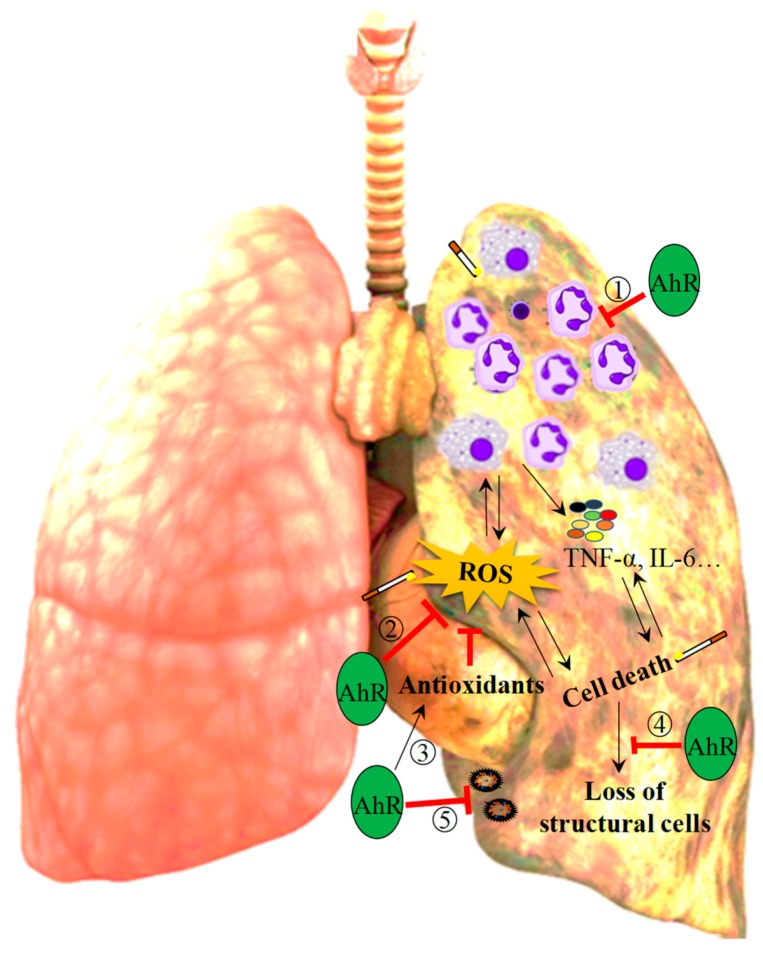
The AhR exerts protective effects in the lungs by attenuating CS-induced lung injury. The AhR attenuates an inter-connected web of pathogenic mechanisms that have been independently established to contribute to the progression of COPD. More specifically, the AhR may contribute to the attenuation of CS-induced inflammation via its role in (1) suppressing CS-induced pulmonary neutrophilia. Additionally, the AhR may attenuate CS-induced oxidative stress via its role in (2) suppressing CS-induced ROS production and (3) promoting the upregulation of anti-oxidants in response to CS. The AhR may also protect against CS-induced lung parenchymal destruction through its ability to (4) reduce the loss of lung structural cells by promoting cellular proliferation and attenuating CS-induced apoptosis and cellular senescence. (5) Finally, the AhR may protect the lungs against bacterial infections that could otherwise trigger COPD-associated exacerbations, such as *S. Pneumoniae* and *P. aeruginosa*, by promoting neutrophil infiltration. Black arrows represent promotion and red bars represent inhibition.

Taken together, there is sufficient evidence to speculate that the AhR may lessen the susceptibility to COPD pathogenesis by attenuating CS-induced pulmonary inflammation, oxidative stress, lung structural cell loss and bacterial infections that can trigger exacerbations (Figure 2). The current body of literature regarding the AhR demonstrates the complexity and often contradictory nature of this signaling pathway and mechanisms associated with disease pathogenesis such as apoptosis, proliferation and senescence. For example, both DRE/XRE binding by ligand-bound AhR as well as AhR-deficiency inhibits cell cycle progression by upregulating p27^KIP1^ [24,147,148,149,150]. Similarly, absence of the AhR as well as exposure to AhR ligands can increase cellular apoptosis [20,151,152,153,154]. These disparities raise an important lingering question: how can the AhR mediate protective effects in some contexts and pathogenic effects in others? Because a key event in AhR-mediated signaling is prolonged AhR degradation following ligand binding [155,156], it is possible that AhR-mediated pathogenicity versus protection in response to TCDD versus CS, respectively, is a consequence of the chronicity of AhR activation- and therefore degradation. This possibility is further strengthened by observations that although TCDD, FICZ and ITE are all high affinity AhR agonists [2], FICZ and ITE are rapidly metabolized and likely non-toxic [157], whereas TCDD is persistent and regarded as highly toxic. Another potential explanation is that there are additional ligand-dependent differences can mediate both pathogenic and protective effects of the AhR in a cell- or context-specific manner. For example, although it is well-established that TCDD-induced pathogenicity is mediated through the canonical AhR signaling pathway, many of the reported protective effects of the AhR against CS-induced lung injury are mediated through non-canonical AhR signaling mechanisms [9,10,11,12,135]. Although there remain disparities in our understanding of AhR signaling, the existing body of literature supports that the AhR is necessary for the maintenance of lung health.
